# High clonal diversity of ESBL-producing *Klebsiella pneumoniae* isolates from clinical samples in a non-outbreak situation. A cohort study

**DOI:** 10.1186/s13756-019-0661-9

**Published:** 2020-01-03

**Authors:** Mariona Xercavins, Elena Jiménez, Emma Padilla, Montserrat Riera, Núria Freixas, Lucia Boix-Palop, Josefa Pérez, Esther Calbo

**Affiliations:** 1 Microbiology Department, CATLAB, Terrassa, Barcelona Spain; 2 Universitat Internacional de Catalunya, C/ Josep Trueta s/n, 08195 Sant Cugat del Vallès, Barcelona Spain; 3 Infectious Disease Unit. Department of Internal Medicine, Hospital Universitari Mutua de Terrassa, Plaza Dr Robert 5, 08221 Terrassa, Barcelona Spain

**Keywords:** ESBL, *Klebsiella pneumoniae*, Clonal diversity

## Abstract

**Background:**

*Klebsiella pneumoniae* has been responsible for a large number of clonal hospital outbreaks. However, some epidemiological changes have been observed since the emergence of CTX-M enzymes in *K. pneumoniae*.

**Aim:**

To analyse the transmission dynamics of Extended Spectrum β-Lactamase-producing *Klebsiella pneumoniae* (ESBL-Kp) in an acute care hospital.

**Methods:**

In 2015 a prospective cohort study was conducted. All new consecutive adult patients with ESBL-Kp isolates in all clinical samples were included. Patients with a previous known infection/colonization by ESBL-Kp and patients in high risk areas (e.g., intensive care units) were excluded. Cross-transmission was defined as the carriage of a clonally-related ESBL-Kp between newly diagnosed patients who shared the same ward for ≥48 h with another case, within a maximum time window of 4 weeks. ESBL-production was confirmed using the double-disk diffusion method and PCR. Clonal relationships were investigated by rep-PCR and multilocus sequence typing (MLST).

**Results:**

Sixty *ESBL-Kp* isolates from 60 patients were included and analysed. Infections and colonizations were classified as hospital-acquired (52%), healthcare-related (40%) or community-acquired (8%).

High genetic diversity was detected. When epidemiological clinical data were combined with the rep-PCR, the patterns identified did not show any cases of cross-transmission. ESBL-Kp were detected in 12.5% of environmental samples. No clonal relationship could be established between environmental reservoirs and patients. The genetic mechanism detected in all strains was associated with *bla*_CTX-M_ genes, and 97% were CTX-M-15.

**Conclusions:**

The dynamics of ESBL-*K. pneumoniae* isolated in our setting could not be explained by clonal transmission from an index patient. A polyclonal spread of ESBL-Kp was identified.

## Introduction

The epidemiology of healthcare-related infections has been characterized in recent decades by the emergence of Gram-negative multidrug-resistant organisms [1]. This increase in resistance appears to be due largely to the production of extended-spectrum β-lactamases (ESBLs) among all Enterobacterales. ESBL-producing *Klebsiella pneumoniae* (ESBL-Kp) is one of the most frequently identified multiresistant pathogens.

*K. pneumoniae* has been responsible for a large number of hospital outbreaks. In the 1990s, these outbreaks were clonal epidemics affecting mainly intensive care patients, and were due to SHV [2] and TEM enzyme types [3]. The first reports of CTX-M *K. pneumoniae* outbreaks were published in the 2000s [4]. Conversely, these CTX-M outbreaks were widespread in general hospital wards and their mortality rates are lower than those previously associated with SHV and TEM outbreaks.

Since the emergence of CTX-M β-lactamases, several clones harboring CTX-M-15 enzymes, often associated with other ESBL types, have been identified [5]. In the case of *K. pneumoniae* it seems that resistance is not restricted to a few genetic backgrounds, and that it is a phenomenon of multiple emergence rather than one involving the spread of a few clones [6]. In fact, high clonal diversity has been reported in non-outbreak [7] and outbreak [8] situations.

In the last 5 years, we have detected an increase in the incidence of hospital-acquired ESBL-Kp infections in our area (from 0.06 in 2011 to 0.35/1.000 stays in 2015) alongside a rise in the prevalence of community-acquired urinary tract infection due to ESBL-Kp (from 2.4% in 2010 to 10.3% in 2014). Most of these clinical isolates harbored CTX M-15 enzymes [9].

With the increase in ESBL-Kp among community-acquired cases [10, 11] and in the hospital setting [4, 12–14] there is a clear need to understand the dynamics of transmission of this relevant pathogen. It is crucial to determine whether the isolation of ESBL-Kp 48 h after hospital admission is actually caused by hospital cross-transmission, and also the extent to which it is preventable and merits infection control interventions.

In this scenario, the aim of the present study was to investigate the dynamics of transmission of ESBL-producing *Klebsiella pneumoniae* by assessing both the clinical epidemiological data and the clonal relatedness of ESBL-Kp among inpatients at a single academic acute care hospital.

## Material and methods

### Setting and study design

In 2015 a prospective cohort study was conducted at Hospital Universitari Mútua Terrassa, Barcelona, a 400-bed acute care hospital with an annual mean number of 97,524 hospital stays for a population of 350,000 inhabitants. Patients are hosted in single or double rooms. Bathrooms are shared in double rooms.

### Inclusion criteria

All new consecutive adult patients with ESBL-producing *Klebsiella pneumoniae* isolates from any specimens obtained by routine clinical practice were included in the study (one sample/patient). Patients with previous known infection/colonization by ESBL-Kp were excluded, as were adult patients admitted to intensive care units (ICU).

### Clinical epidemiological data collection and infection control standards

Definitions:

- “Index patient” was defined as an inpatient with a newly recognized clinical sample yielding ESBL-Kp.
- “Contact patient” was defined as a person who shared the same room for > 24 h with an index patient without initiation of contact precautions.
- “Cross-transmission” was defined as the carriage of a clonally-related ESBL-Kp among newly diagnosed patients sharing the same room or ward for ≥48 h with another index case, within a maximum time window of 4 weeks.
- “Healthcare relation” was defined according to *Friedman* et al. [15] “Hospital-acquired infection” was defined as an infection acquired during hospital care that was not present or incubating at admission (infections occurring 48 h after admission were considered) or in a patient discharged from hospital in the previous 30 days. “Healthcare-related infection” was diagnosed if the patient fulfilled at least one of the following criteria: (i) having resided in a nursing home or long-term care facility in the 30 days before the episode; (ii) hospitalized in an acute care hospital for ≥48 h in the 90 days before the episode; (iii) having attended a hospital or hemodialysis clinic or received intravenous therapy in the 30 days before the episode; and/or (iv) having received intravenous therapy, wound care, enteral nutrition or healthcare at home in the 30 days before the episode. Otherwise, the infection was classified/considered as community-acquired.

All index patients were placed on contact precautions. Index patients were screened at the time of first detection in order to determine colonization. Screening samples included a rectal swab and urine sample in patients with a Foley catheter. According to ESCMID guidelines [16], three or more repeatedly negative screening cultures over the course of one or 2 weeks in a patient who had not received antimicrobial therapy for several weeks were needed to consider that a patient was decolonized and, therefore, that contact precaution measures could be suspended. No active decolonization policies were conducted. Contact precautions included a single-patient room and the use of gloves and gowns by healthcare workers.

Basic infection control standards included proper hand hygiene (as indicated in the WHO guidelines) [17]. In 2015, compliance with a hospital-wide project promoting hand hygiene was 64%. During the study period a stringent environmental cleaning process including twice-daily hospital cleaning with detergents, as well as enhanced terminal cleaning of rooms of targeted patients on contact precautions, was also conducted.

Infection control staff routinely visited all inpatients with colonization or infection due to ESBL-producing *Klebsiella pneumoniae*. Data on demographics, type of sample, healthcare-relatedness, time from admission until ESBL-Kp identification and movements around the hospital (including detailed information regarding rooms and wards occupied during the hospital stay) were prospectively collected as part of the standard epidemiological clinical work-up conducted by the infection control team. Rectal swab screening for contact patients was also performed as well. All patients with known ESBL carriage were screened whenever they were readmitted to the hospital as part of standard infection control practices. No other active surveillance was applied.

### Environmental samples

A surveys of environmental colonization of ESBL-Kp were performed in some of the rooms occupied by ESBL-Kp colonized or infected patients during the year under study. Four samples were obtained in each surveyed room (tap of the sink, surface around the sink, bedpan and bedpan washer tap) [18].

Samples were obtained for culture by rubbing gauzes moistened with thioglycolate broth repeatedly over designated sites in the immediate vicinity of the patient environment and they were stored in screw-cap sterile containers with 10 mL of thioglycolate broth. The containers were incubated for 24 h at 37 °C and then inoculated onto ChromID ESBL (bioMérieux) [4, 19].

### Microbiological methods

Bacterial identification and susceptibility testing was performed using Vitek2 System (BbioMérieux). EUCAST breakpoints were used for interpretation of the results. ESBL-production was confirmed using the double-disk diffusion-method. ESBL characterization was performed by commercial PCR (Check-MDR CT103XL, Hain).

The genetic relationship between all 60 ESBL-Kp isolates was determined by automated repetitive-sequence-based PCR using the Diversilab system (bioMérieux), following the manufacturer’s recommendations. Rep-PCR fingerprinting profiles were compared and analyzed by Diversilab (version 3.6) software using Pearson correlation coefficient pairwise pattern matching and the unweighted pair group method with arithmetic mean (UPGMA) clustering algorithm. The cutoff value for similarity in order to establish strain identity was 95%.

Multilocus sequence typing (MLST) was performed using seven conserved housekeeping genes (*gapA, infB, mdh, pgi, phoE, rpoB* and *tonB*) [20]. The protocol of the MLST procedure, including allelic type and sequence type (ST) assignment methods, is available from the MLST databases of the Pasteur Institute (Paris, France) http://bigsdb.pasteur.fr/klebsiella/klebsiella.html. The phylogenetic relationships between the different ST found in the study were established by Phyloviz (https://online.phyloviz.net/index) using the goeBURST algorithm.

## Results

During 2015, 60 consecutive index cases were identified. Demographic and clinical data of patients and isolates are shown in Table 1. In order of frequency, the origins of the clinical samples were urine (47, 78%), surgical wounds (six, 10%), blood (six, 10%) and respiratory samples (one, 2%). Thirty-two isolates were obtained from patients admitted to the hospital emergency department, 16 in surgical wards and 12 in medical wards.

**Table 1:** Demographics and clinical characteristics of patients and isolates

Patients characteristics	Community- acquired	Healthcare-related	Hospital- acquired infection	Total
Number of isolates and patients	5	24	31	60
Gender, male (N)	1	12	20	33
Sample sites (N)				
- Urine	5	21	21	47
- Wounds	0	1	5	6
- Blood	0	2	4	6
- Respiratory	0	0	1	1
Units (N)				
- Surgery wards	1	2	13	16
- Medical wards			12	12
- Emergency department	4	22	6	32

**Fig. 1:**
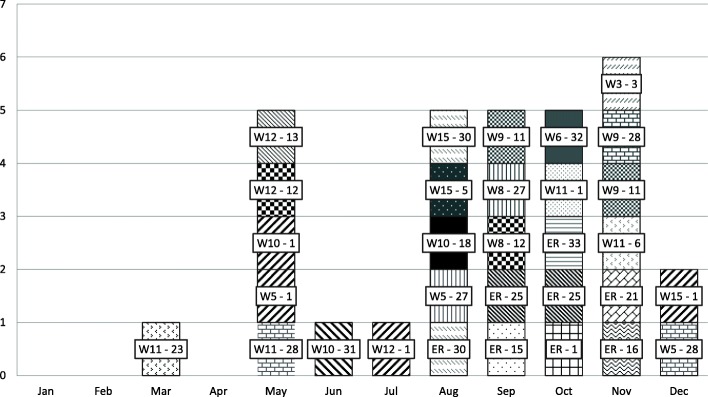
Rep-PCR pattern of ESBL-*K. pneumoniae* isolates of hospital-acquired infection origin per month and ward. Each square represents the number of isolates; every rep-PCR pattern is represented by a different drawing and number (1–33). All the isolates are labelled according to the ward (W: 5 to 15) or ER (Emergency room) and rep-PCR pattern (number)

New index cases were detected with a median frequency of 2.5 (range 0–6) patients per month and there were no outbreaks in any specific hospital area (Fig. 1). Counting all *K. pneumoniae* isolates, the proportion of samples with ESBL-producing enzymes in our hospital in 2015 was 26.10%, compared with 27.52% in Catalonia as a whole [21].

Among the clinical samples, 47 (78.3%) were interpreted as infections and 13 (21.7%) as colonizations.

Hospital-acquired infection/colonization was demonstrated in 31 index cases (52%), healthcare-related infection/colonization in 24 (40%) patients, and community-acquired infection/colonization in five (8%). Among healthcare-related samples, 11 (17%) were from nursing home residents. At two particular nursing homes two cases were identified, but neither a temporal nor a genetic relationship could be established between the isolates in either setting.

**Fig. 2:**
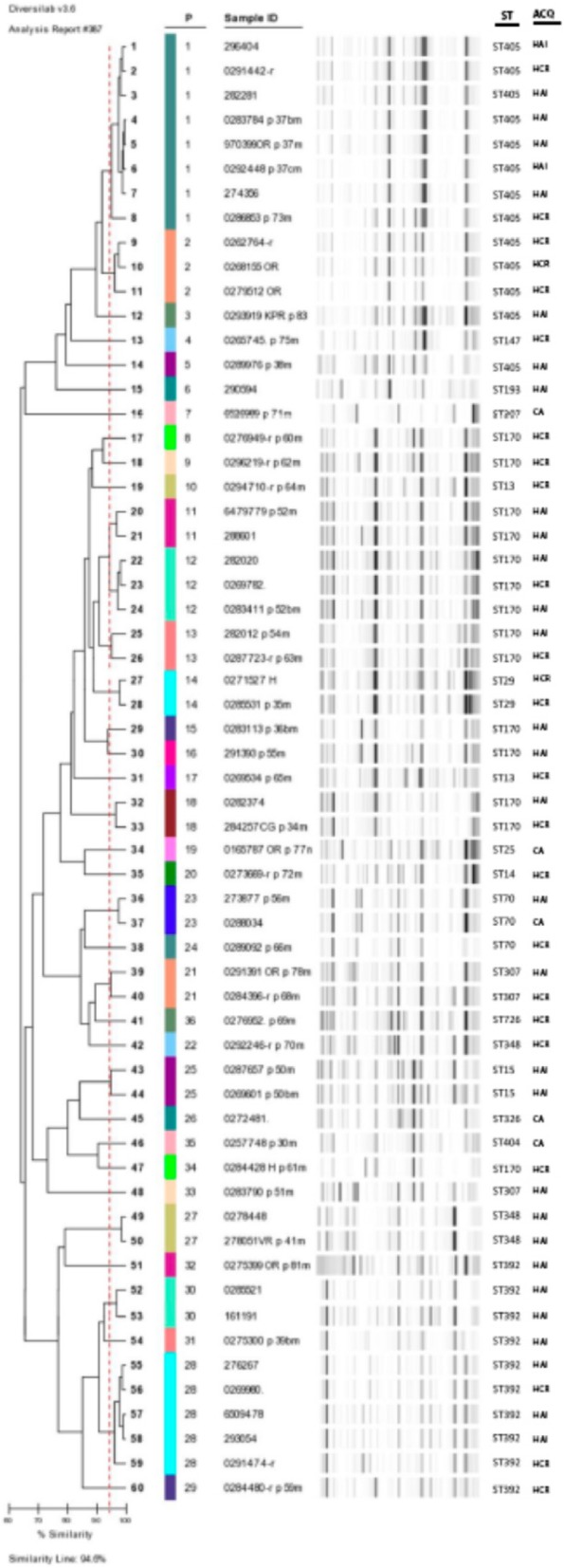
Cluster analysis and virtual gel image from DiversiLab generated fingerprints of the 60 *K. pneumoniae* strains, including corresponding ST results from MLST. ACQ: Site of acquisition/HAI:hospital-acquired infection/HCR:healthcare-related/CO:community-acquired

**Fig. 3:**
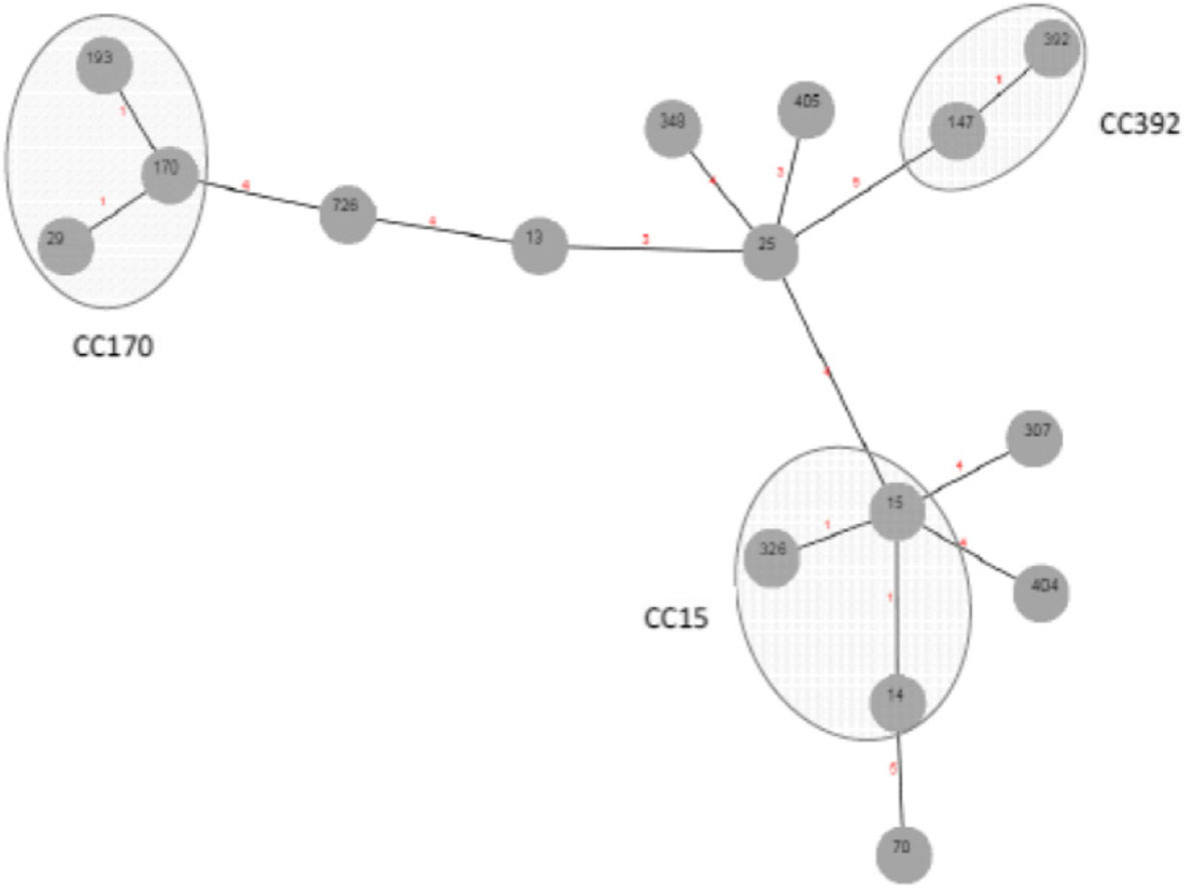
Phylogenetic relationships between the STs detected in our study. ST29 and ST193 are single-locus variant of ST170 as well as, ST 326 and ST14 are single-locus variant of ST15. Numbers circled indicate the ST and the number on the branch indicates the number of different alleles between STs. CC: clonal complex

High genetic diversity was detected. The isolates were classified into 36 different patterns (rep-PCR, Diversilab); only 4/36 patterns included three or more isolates. Sixteen sequence types (ST) were identified. The most prevalent STs encountered were ST170 (23%), ST405 (21%) and ST392 (16%). Altogether, these STs represented 60% of the isolates. A summary of the characterization of the 60 isolates is shown in Fig. 2, and the phylogenetic relationships between the different STs are shown in Fig. 3. See Additional files 1, 2 and 3, available as Supplementary information, with the characteristics of each isolate according the site of acquisition.

Thirty-one strains with 19 different rep-PCR patterns and eight different STs were identified among hospital-acquired infection samples. The most frequent were ST170 (26%), ST405 (26%) and ST392 (23%).

Among healthcare-related cases, 24 strains were collected with 22 different patterns identified by rep-PCR and 11 STs. In this case the most frequent were ST170 (25%), ST405 (21%) and ST392 (13%).

Five community-acquired samples were included which showed five different patterns by rep-PCR and MLST. Two community-acquired sequence types (ST70 and ST307) were also found in two healthcare-related strains and in three hospital-acquired infection strains.

No cases of cross-transmission were found when epidemiological clinical data were combined with the rep-PCR patterns identified (Fig. 1).

The genetic mechanism detected in all ESBL-Kp isolates was associated with the presence of *bla*_CTX-M_ genes. Most of the CTX-M detected belonged to group 1: CTX-M-15 (58 strains) and CTX-M-32 (one strain). One strain belonged to group 9. No carbapenemase producers were detected. All ESBL enzymes in hospital-acquired and community-acquired samples were CTX-M-15, as were 22 out of 24 identified in healthcare-related strains (the exceptions being one CTX-M-9 and one CTX-M-32).

Antibiotic susceptibility testing showed that all isolates were resistant to cefotaxime and ceftazidime. The antimicrobial resistance pattern is summarized in Table 2. The phenotypical characterization showed differences between isolates with the same sequence type.

**Table 2:** Antimicrobial resistance of *K. pneumoniae* ESBL-producing isolates

Antibiotic	Community- acquired (*N* = 5)	Healthcare- related *N* = 24	Hospital- acquired infection (*N* = 31)	Total
Amoxicillin-clavulanic acid	3/5	21/24	31/31	55/60
Amikacin	0/5	2/24	1/27	3/57
Cefepime	5/5	24/24	31/31	60/60
Cefuroxime	5/5	24/24	31/31	60/60
Cefotaxime	5/5	24/24	31/31	60/60
Ceftazidime	5/5	24/24	31/31	60/60
Ciprofloxacin	3/5	21/24	31/31	55/60
Ertapenem	0/5	0/24	0/31	0 / 60
Gentamicin	1/5	13/24	14/31	28 /60
Imipenem	0/5	0/24	0 /31	0 /60
Piperacillin/tazobactam	2/5	12/24	20/31	34/60
Trimethoprim/sulfamethoxazole	3/5	21/24	29/31	53/60

### Environmental samples isolated

ESBL-Kp were detected in 4/32 (12.5%) environmental samples from three rooms.

One room had two positive samples (surface around the sink and bedpan washer tap) with an identical rep-PCR pattern, though it was not identified with a particular patient.

The other positive samples were isolated in two different rooms. The first one was on the surface around the sink with the same rep-PCR pattern as the strain isolated in the previous occupant of the room; the second was cultured from a sink tap and presented a rep-PCR pattern different from the one identified in a patient who had previously occupied the room.

## Discussion

In a non-outbreak setting, no cases of cross-transmission of ESBL-producing *Klebsiella pneumoniae* could be demonstrated in general wards (non-ICU) in our hospital during the year under study. A high genetic diversity was confirmed by both rep-PCR and MLST.

Traditionally, ESBL-*Kp* cross-transmission via the hands of healthcare workers [2] and the lower gastrointestinal tract of colonized patients [22] has been documented as the main reservoir of these microorganisms during hospital outbreaks [5, 23]. However, in our setting, we could not demonstrate either a clonal or a clinical epidemiological relatedness between consecutive non-duplicate ESBL-*Kp* strains isolated during 2015. Therefore, this traditional dynamics cannot explain our epidemiology. A low rate of hospital-acquired infection transmitted by highly drug-resistant Gram-negative bacteria was also found in a large multicenter trial involving 18 Dutch hospitals, and in a single-center Swiss study of ESBL-producing Enterobacterales [24, 25].

Interestingly, a recent study showed that only half of the cases of healthcare-acquired infection or colonization due to multi-drug resistant organisms (MDRO) according to CDC definitions are truly hospital-acquired [26]. Similarly, some reports suggest that some patients with infections caused by ESBL-producing *K. pneumoniae* isolates seen at hospitals should be epidemiologically defined as community-associated [9, 11]. Therefore, in these scenarios, ESBL-producing *K. pneumoniae* is more likely to have been imported into the hospital than to have originated there. In fact, hospital outbreaks originating from a community source of ESBL-producing *K. pneumoniae* have already been described [4].

However a word of caution is in order before concluding that there is no transmission. Carriership is generally asymptomatic and universal screening is not conducted at our hospital. As a result, intermediate patients may be missed and no epidemiological link can be made. In addition, a seasonal variation was identified in our study. Nevertheless, it was recently shown that in a non-outbreak setting, importation of ESBL-producers into hospitals seems to be at least as frequent as transmission events during the hospital stay [27]. The total lack of clonal relatedness between index cases and contact patients makes cross-transmission highly improbable in our setting. It should also be stressed that for financial reasons universal screening for all MDRO is not carried out at most acute care hospitals.

Hospital environmental contamination has been reported as the source of several ESBL-Kp outbreaks [4, 28, 29]. In our study, no clonal relationship with environmental samples could be established. However, it is conceivable that more extensive environmental screening would have identified a reservoir possibly missed by the present design.

Only cross-transmitted MDRO are preventable and are reasonable targets for an infection control program. Non-preventable events may be related to selective antibiotic pressures that trigger the emergence of ESBL-producing *K.pneumoniae* colonizing the gastrointestinal tract after admission. In this scenario, infection control measures must be coordinated with antimicrobial stewardship programs to stop the endemic evolution of ESBL-producing *K. pneumoniae*.

Regarding the antibiotic resistance phenotype, isolates were mostly classified as CTX-M 15 producers. These results are in agreement with those previously reported in other countries and thus corroborate the wide distribution of this enzyme [12, 30–33].

The STs identified in our setting belong to previously described international clones associated with multidrug-resistant *K. pneumoniae* isolates. ST170 was the most frequent sequence type identified in our cohort. To our knowledge, this is the first report of ST170 in human strains. Moreover, ST170 was only detected in hospital strains without any epidemiological relationship, suggesting the possibility of an endemic situation. The other two frequently identified STs in our cohort (ST405 and ST392) have been described elsewhere in Europe and in South America in strains of human origin*.*

Machuca et al. [34] published the first report of a *K. pneumoniae* ST405 without harbouring a carbapenemase, the type we found in our isolates. Previously, ST405 has been described as a clone capable of disseminating different quinolone and beta-lactam resistance determinants (including ESBL and carbapenemase): in Spain and France among OXA-48 and CTX-M15- producing isolates, and in Yemen among NDM and CTX-M-15 producers. ST392 has been reported in *K. pneumoniae* in CTX-M-15 associated with carbapenemase: in KPC in China, and in OXA-48 in Europe. This is the first time that ST392 has been described in *K. pneumoniae* carrying only CTX-M.

The phenotypic method, consisting in the identification of species type and resistance towards several selected antibiotics, was unable to detect ST or rep-PCR groups. This suggests that the phenotypic method is not suitable for infection control procedures and that molecular identification is crucial for the definition of cross-transmission. Similar results were published by Souverein et al. [8].

This study has some limitations. First, the single-center study design may limit the generalizability to other settings and we cannot rule out the possibility that the lack of transmission at our institution may be attributable to the high level of infection control and to the low number of beds per room.

Second, no plasmid typing was performed. The criteria for cross-transmission in the present study did not address the possibility of horizontal transmission of common plasmids between different Enterobacterales species [6, 35].

Third, the gold standard assay for molecular typing is pulsed-field gel electrophoresis (PFGE), due to its high discriminatory power. The discriminatory power of rep-PCR is generally similar to that of PFGE. PCR methods are preferable in the study of small, time-limited outbreaks, while in complex outbreaks of longer duration, in which clonal evolution and dynamics are studied, PFGE should be used. Molecular typing methods based on DNA sequencing such as MLST are applicable in global epidemiological studies [36]. The initial assessment in our study was made using the rep-PCR, and the MLST method confirmed the diversity in our population.

Fourth, the lack of systematic active surveillance of all inpatients admitted or discharged from hospital may have meant that some transmission events were missed. However, systematic surveillance of all contact patients did not demonstrate cross-transmission in this high-risk situation. Fifth, the detection method, i.e., screening for colonization merely by collecting rectal swabs without any enrichment to increase the detection sensitivity, may have missed some ESBL-KP strains in contact patients. Finally, since we only studied ESBL-Kp in a non-outbreak scenario, it may not be possible to extrapolate our results to other Enterobacterales or to other epidemic settings.

In conclusion, in this epidemiological study of a non-outbreak setting, we identified a polyclonal spread of ESBL-Kp with high genetic diversity. Neither clonal cross-transmission nor environmental reservoirs could be demonstrated. Our data suggest that the probable importation of ESBL-Kp into the hospital may explain the dynamics of hospital-acquired cases in our setting. These findings question the validity of the contact precaution measures applied to control ESBL-Kp epidemics. More studies are now required to explore this matter further.

## Supplementary information

**Additional file 1.** Characteristics community-acquired isolates.

**Additional file 2.** Characteristics healthcare-related isolates.

**Additional file 3.** Characteristics hospital-acquired isolates.

## Data Availability

Not applicable.

## Abbreviations

CDC: Centers for Disease Control and Prevention
ESBL: Extended-spectrum β-lactamases
ESBL-Kp: Extended-spectrum β-lactamases-*Klebsiella pneumoniae*
ESCMID: European Society for Clinical Microbiology and Infectious Diseases
EUCAST: European Committee on antimicrobial susceptibility testing
ICU: Intensive care unit
MDRO: Multi-drug resistant organisms
MLST: Multilocus sequence typing
PCR: Polymerase chain reaction
ST: Sequence type
WHO: World Health Organization
